# Supplementary material for: Longitudinal Consumption of Ergothioneine Reduces Oxidative Stress and Amyloid Plaques and Restores Glucose Metabolism in the 5XFAD Mouse Model of Alzheimer’s Disease
Source: Pharmaceuticals (Basel). 2022 Jun 13;15(6):742. doi: 10.3390/ph15060742 (PMC9228400; doi:10.3390/ph15060742)

pharmaceuticals

MDPI

Article

Longitudinal Consumption of Ergothioneine Reduces Oxidative Stress and Amyloid Plaques and Restores Glucose Metabolism in the 5XFAD Mouse Model of Alzheimer's Disease

Clayton A. Whitmore [1,2], Justin R. Haynes [1,2], William J. Behof [1,2], Adam J. Rosenberg [1,2], Mohammed N. Tantawy [1,2], Brian C. Hachey [3], Brian E. Wadzinski [4], Benjamin W. Spiller [4], Todd E. Peterson [1,2], Krista C. Paffenroth [4,9], Fiona E. Harrison [5,9,13], Robert B. Beelman [6], Printha Wijesinghe [7], Joanne A. Matsubara [7] and Wellington Pham [1,2,8,9,10,11,12,13,*]

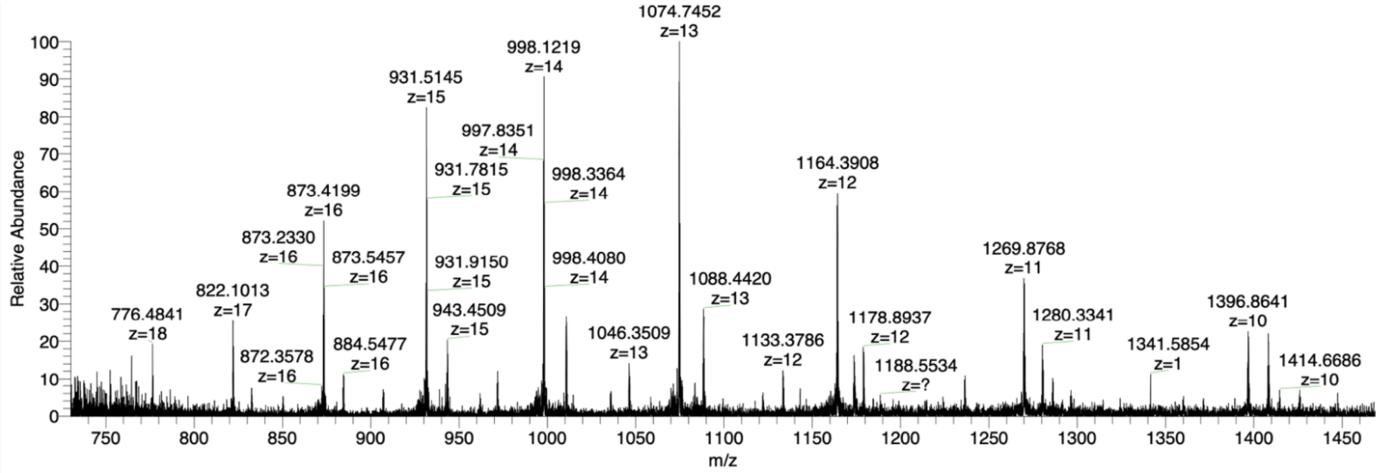

Figure S1. Orbit trap mass spectrometry to characterize the eluting His-tag GFP nanobody (14 kDa) from the 2D-HPLC.

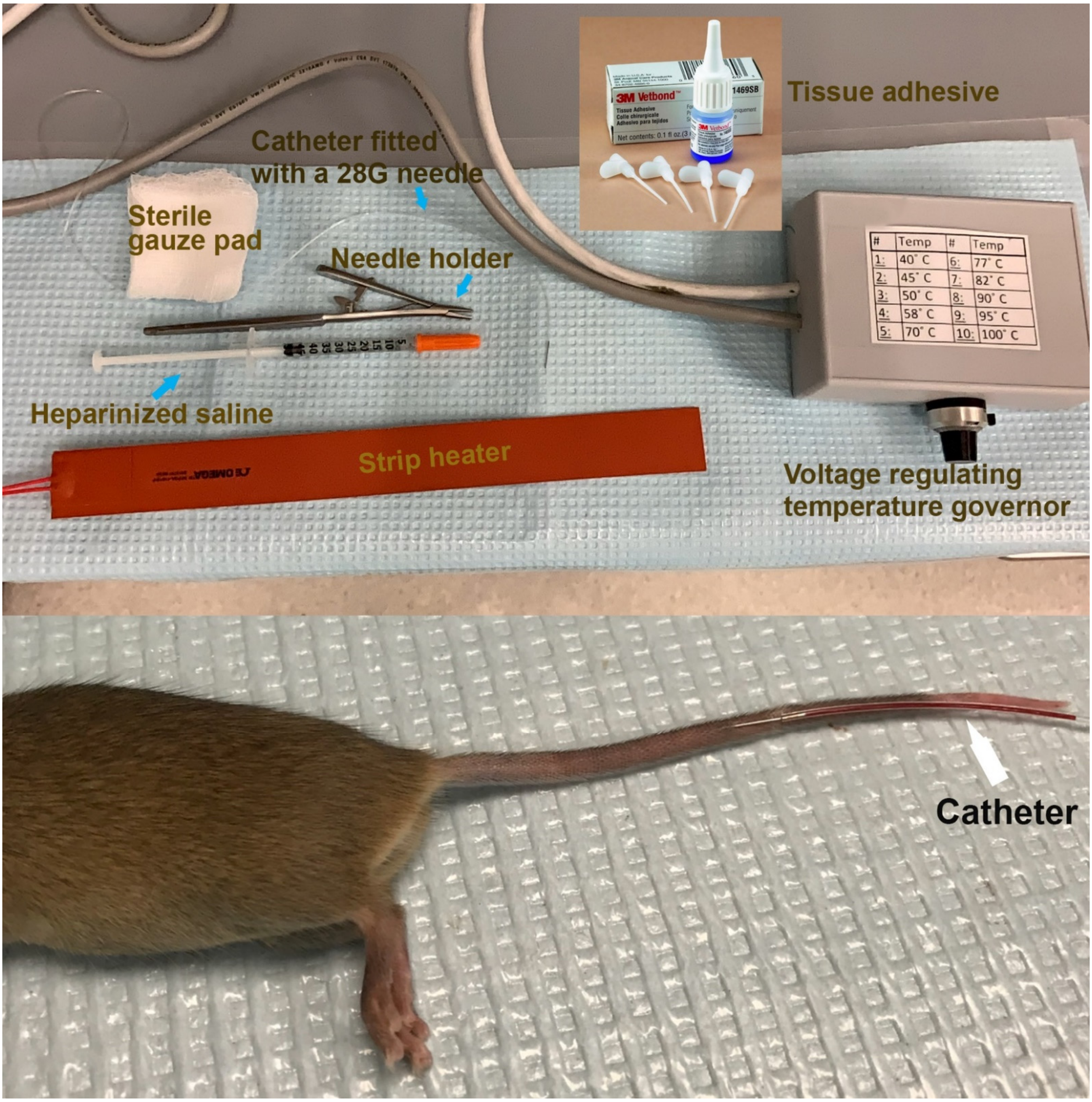

Figure S2. Tools and supplies are used for the injection of the PET radioligands via the tail vein. The lateral tail vein of the anesthetized (2.5% isoflurane) animal is warmed with a strip heater before insertion of the heparinized saline-filled catheter. After successfully generating the catheter, the needle is glued with a small drop of tissue adhesive prior to injection. After injection, the void volume of the probe in the catheter is displaced by heparinized saline.

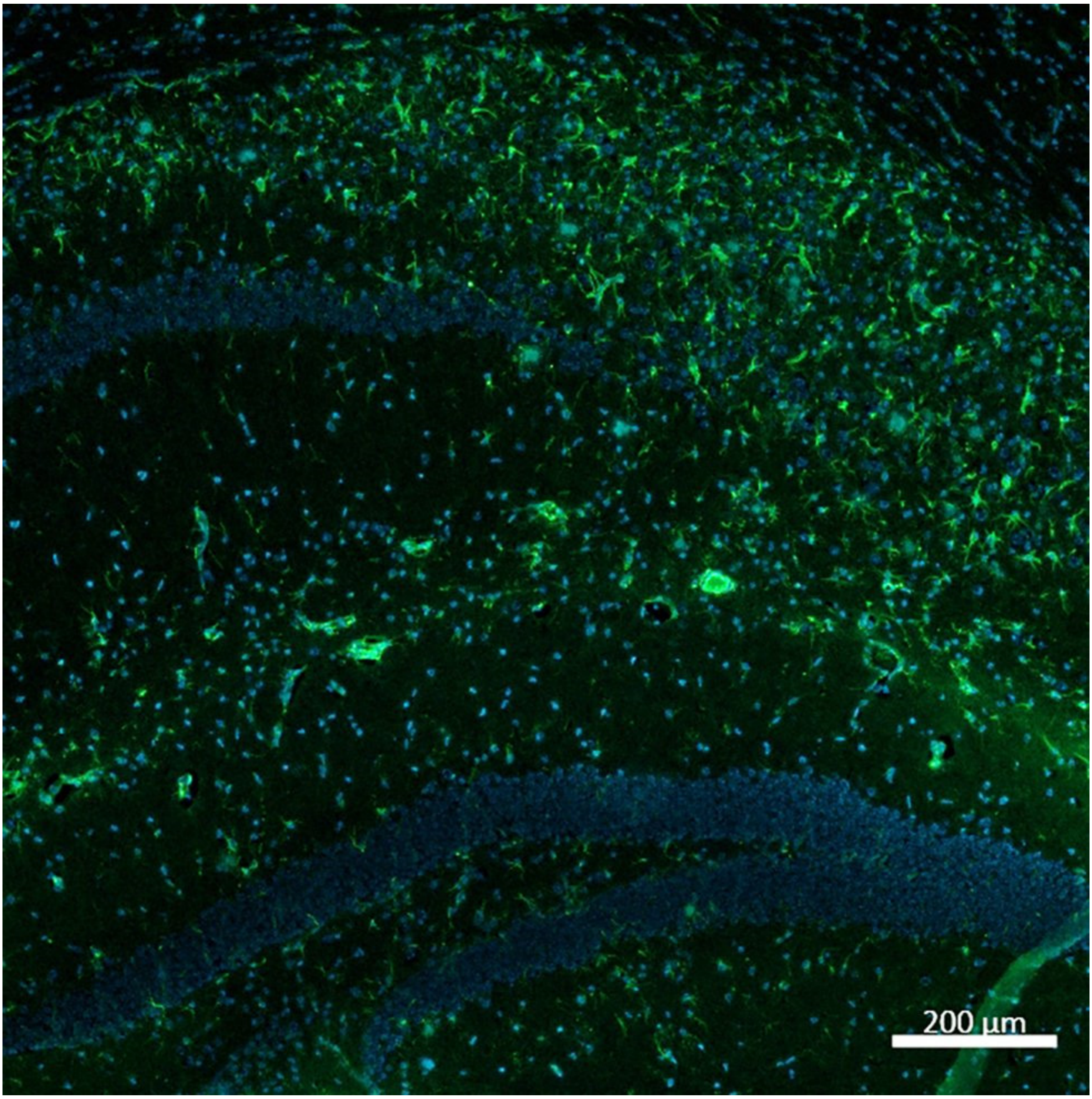

Figure S3. Stereological analysis of immunohistochemistry data using ImageJ. This is a sample work to quantify the pixel counts of GFAP on a non-treated 5XFAD.

To ensure precise analysis, all data should be obtained in the same region, with the same scale bar dimension. For instance, the hippocampus in this work showing the high-resolution digital images of the whole slides at 10x magnification to a resolution of 2.6 µm × 2.6 µm per pixel using the Axio Observer microscope:

Using imageJ to measure the scale bar and calibrate the figure dimension in micrometers. Once it is appropriately performed, the figure now would look like this:

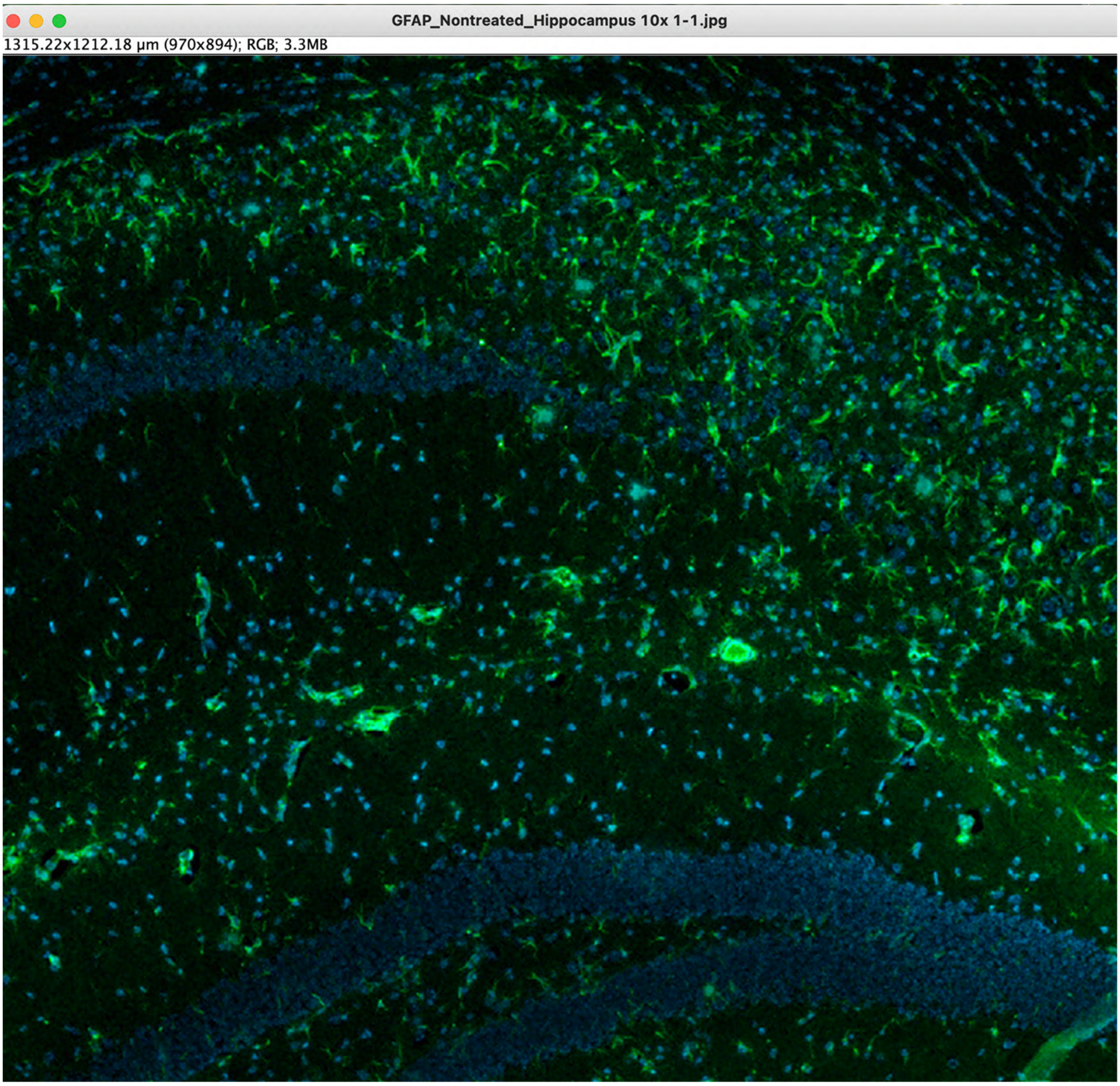

Then, the scale bar is removed prior to extracting the information of the GFAP-positive astrocytes by separating the data into each individual channel:

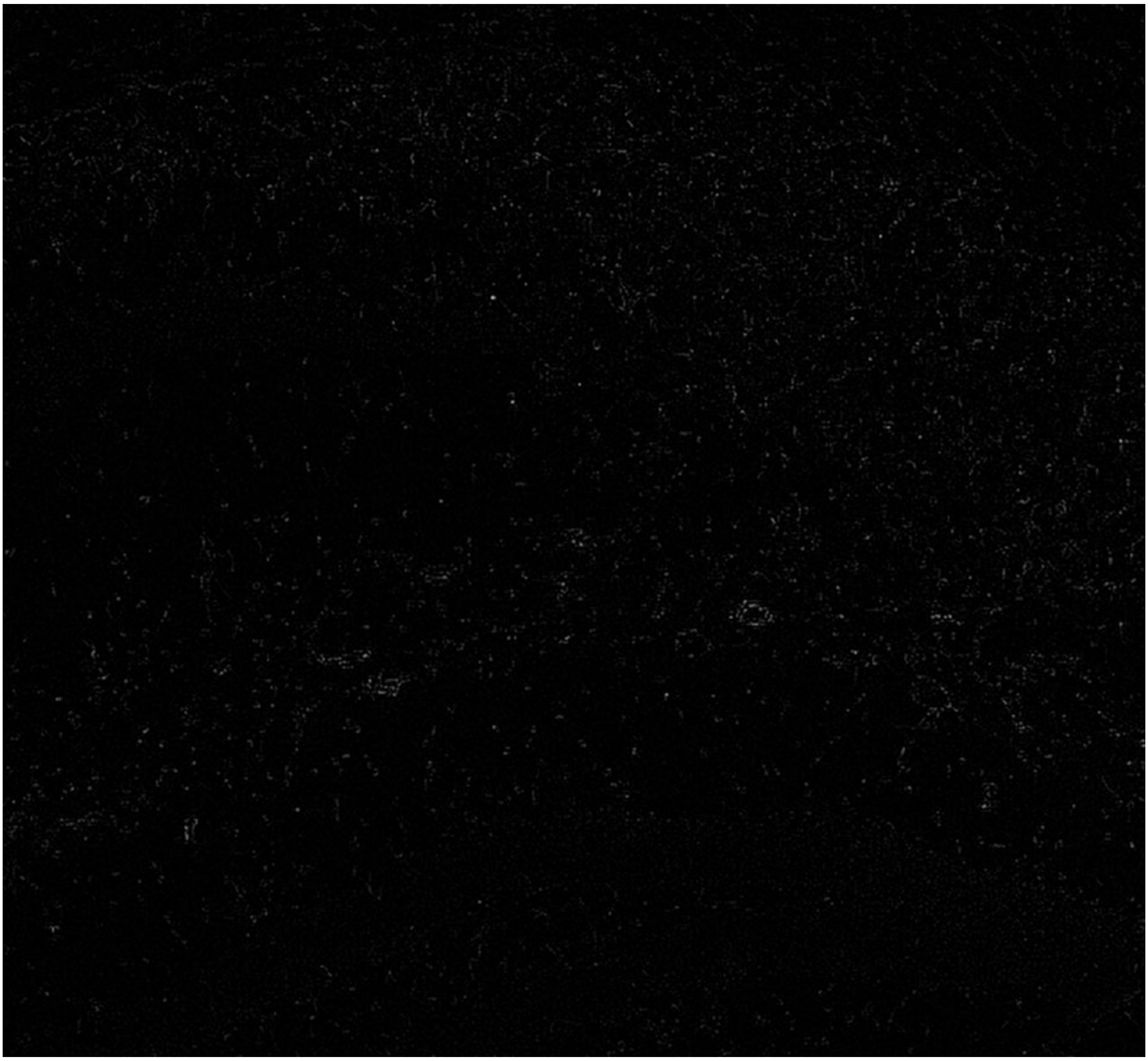

Red channel.

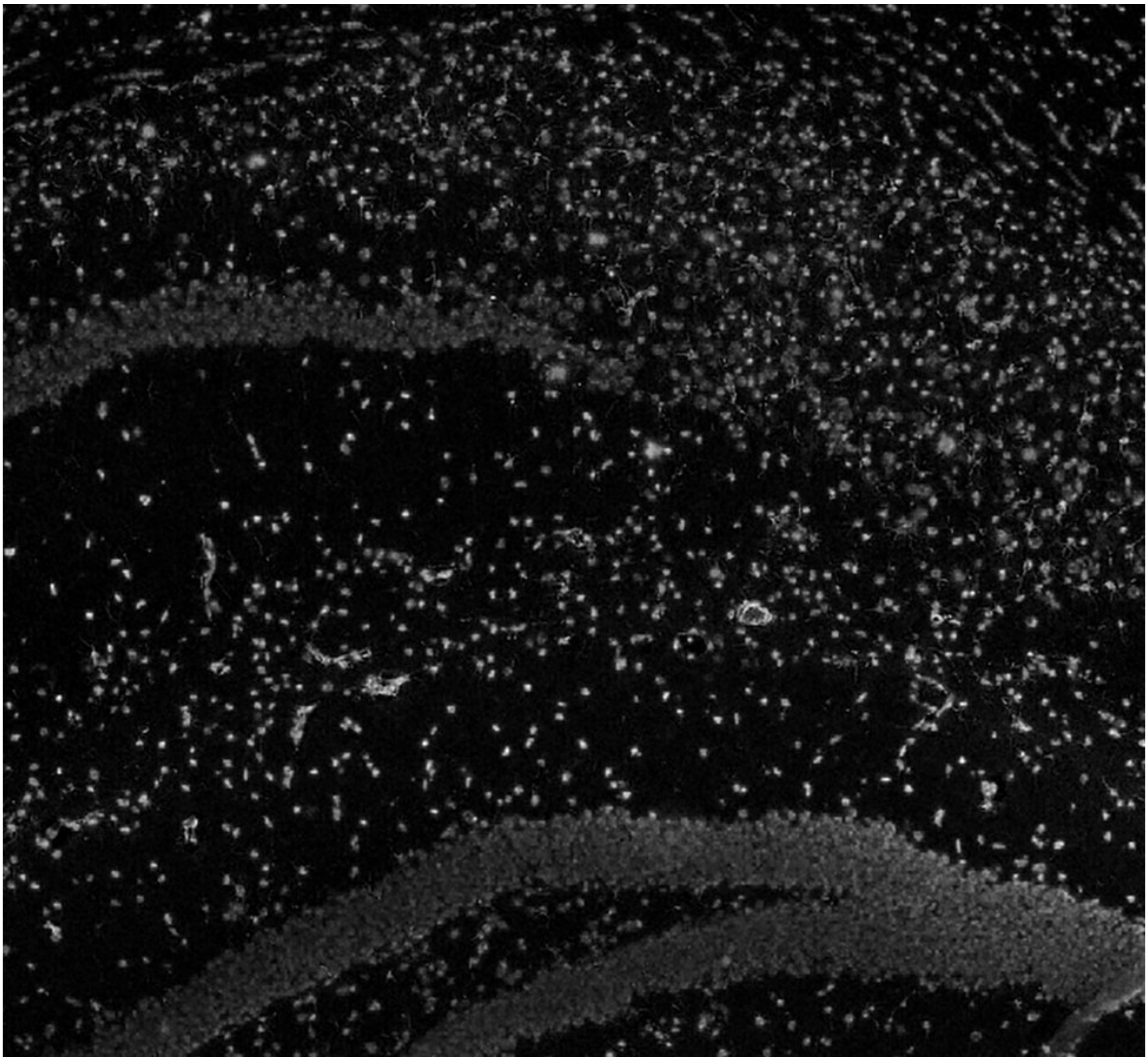

Blue channel.

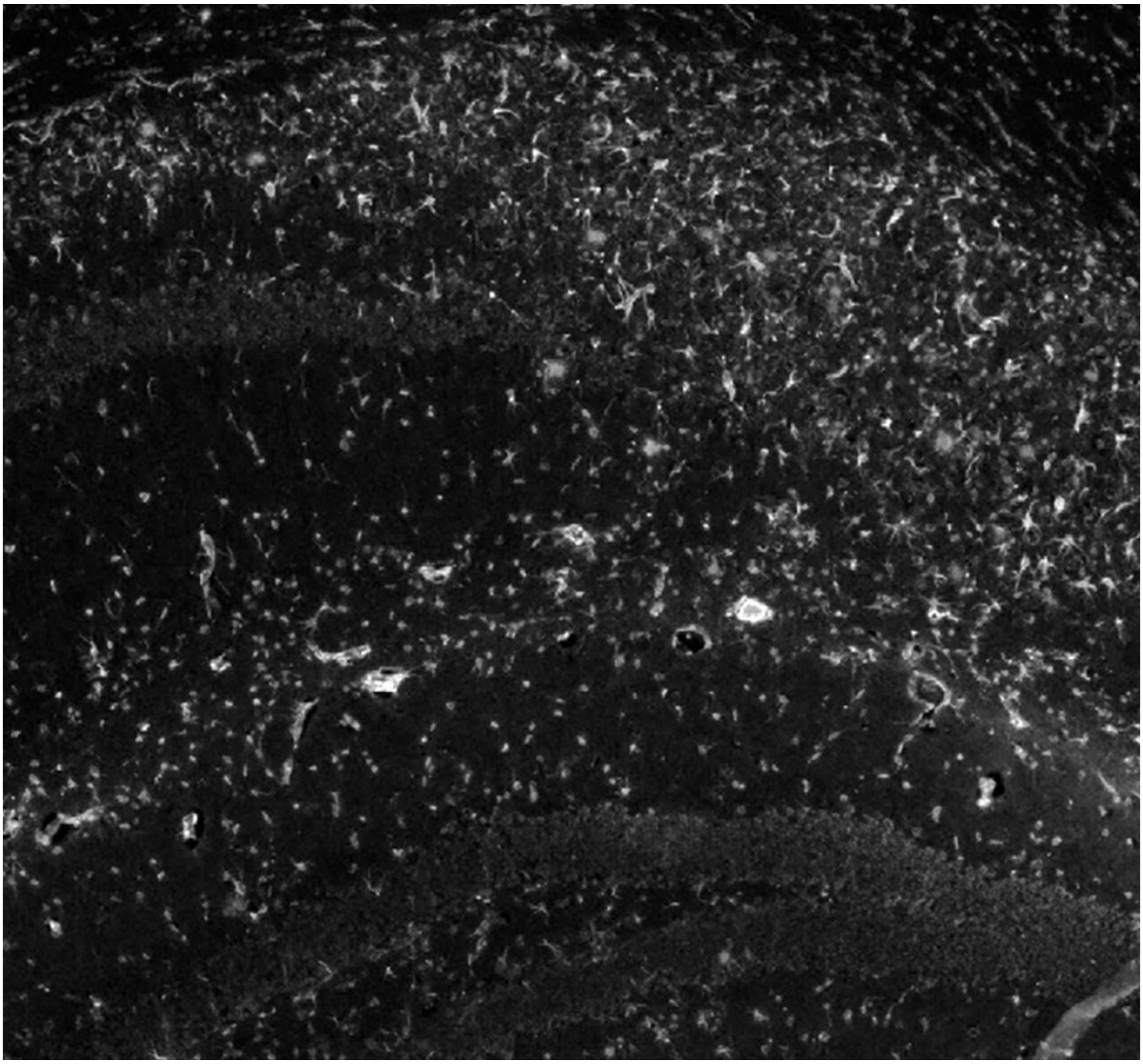

Green channel.

The green channel is associated with astrocytes, and thus, only these data were proceeded forward for thresholding and counting:

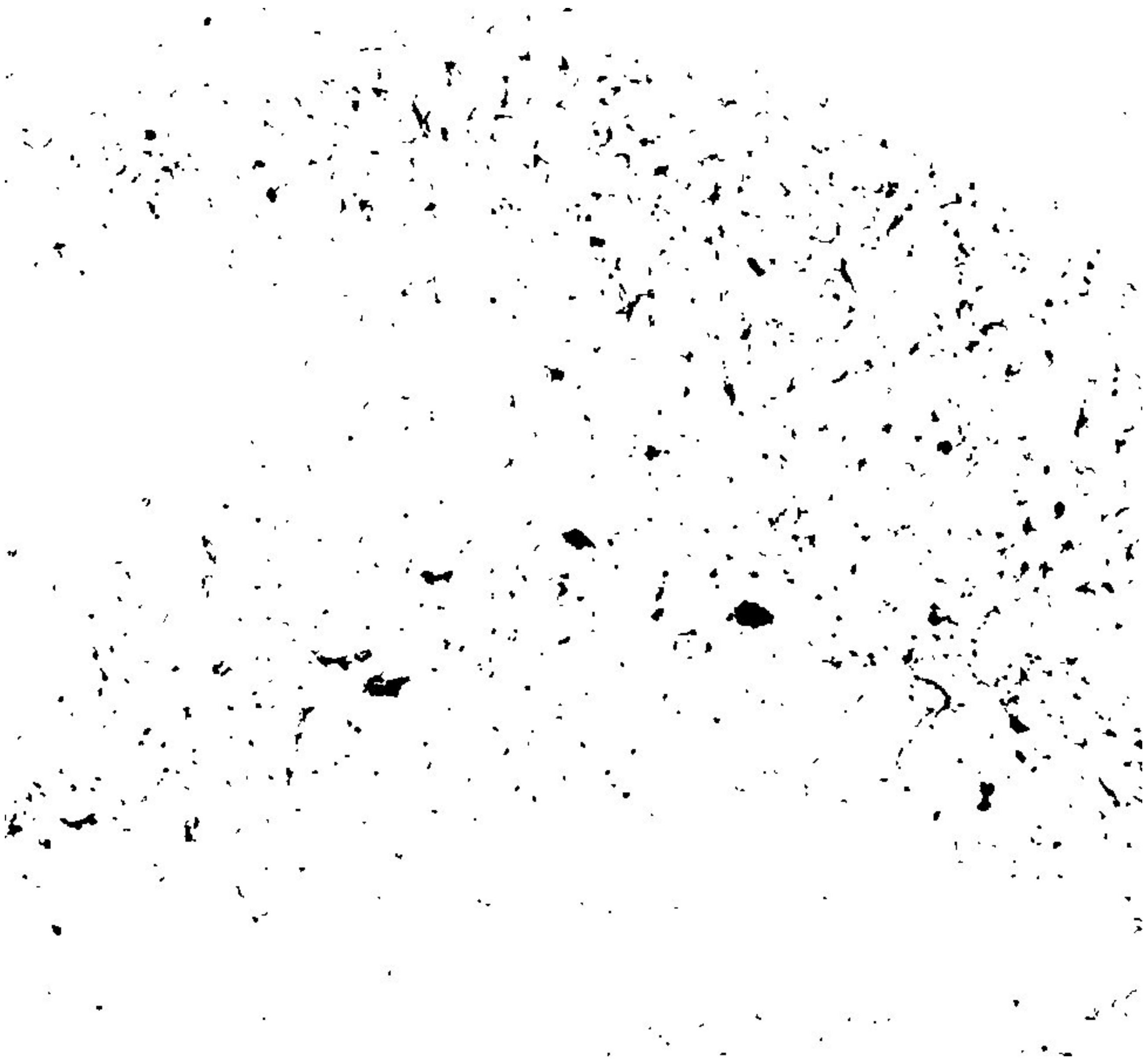

Supplement: Supplementary file 1 [file pharmaceuticals-15-00742-s001.zip › pharmaceuticals-1737460-supplementary.pdf]
